# Prognostic Value of Pretherapeutic Primary Tumor MTV from [^18^F]FDG PET in Radically Treated Cervical Cancer Patients

**DOI:** 10.3390/metabo11120809

**Published:** 2021-11-28

**Authors:** Paulina Cegla, Frank Hofheinz, Witold Cholewiński, Rafał Czepczyński, Anna Kubiak, Jörg van den Hoff, Agnieszka Boś-Liedke, Andrzej Roszak, Ewa Burchardt

**Affiliations:** 1 Department of Nuclear Medicine, Greater Poland Cancer Centre, 61-866 Poznań, Poland; witold.cholewinski@wco.pl; 2 Helmholtz-Zentrum Dresden-Rossendorf, Institute of Radiopharmaceutical Cancer Research, 01328 Dresden, Germany; hofheinz@hzdr.de (F.H.); j.van_den_hoff@hzdr.de (J.v.d.H.); 3 Department of Electroradiology, Poznan University of Medical Sciences, 61-701 Poznań, Poland; andrzej.roszak@wco.pl (A.R.); ewa.burchardt@wco.pl (E.B.); 4 Department of Endocrinology, Metabolism and Internal Diseases, Poznan University of Medical Sciences, 61-701 Poznań, Poland; czepczynski@ump.edu.pl; 5 Department of Nuclear Medicine, Affidea Poznan, 61-485 Poznań, Poland; 6 Greater Poland Cancer Registry, Greater Poland Cancer Centre, 61-866 Poznań, Poland; anna.kubiak@wco.pl; 7 Department of Macromolecular Physics, Adam Mickiewicz University, 61-614 Poznań, Poland; agnbos@amu.edu.pl; 8 Department of Radiotherapy and Gynaecological Oncology, Greater Poland Cancer Centre, 61-866 Poznań, Poland

**Keywords:** positron emission tomography/computed tomography, cervical cancer, [^18^F]FDG, metabolic parameters

## Abstract

The aim of this study was to assess the usefulness of pretherapeutic primary tumor metabolic tumor volume (MTV) in the prognosis of radically treated cervical cancer patients. Retrospective, single-centre analysis was performed on a group of 508 cervical cancer patients. All patients underwent a pretreatment [^18^F]FDG PET/CT study for the assessment of the disease stage. Several PET-derived parameters—namely, maximum standardized uptake value (SUV_max_), mean standardized uptake value (SUV_mean_), total lesion glycolysis (TLG) and MTV, as well as the clinical parameters, were analysed in terms of the overall survival (OS), event-free survival (EFS), locoregional control (LRC) and freedom from distant metastases (FFDM). Hyperthermia and brachytherapy were prognostic for EFS, OS, and LRC.FIGO stage > II showed a significant effect on EFS, OS, and FFDM. Moreover, hysterectomy was prognostic for OS and histology was prognostic for FFDM. From the PET-derived parameters only MTV of the primary tumor had a significant influence on OS (cutoff point: >12.7 mL, HR: 2.8, 1.75–4.48 95% CI, *p* < 0.001), LRC (cutoff point: >13.7 mL, HR 2.82, 1.42–5.61 95% CI, *p* = 0.003), EFS (cutoff point: >10.4 mL, HR: 2.57, 1.67–3.97 95% CI, *p* < 0.001) and FFDM (cutoff point: >10.4 mL, HR: 5.04, 1.82–13.99 95% CI, *p* = 0.002). Pretreatment MTV from the primary tumor is the only independent prognostic parameter in OS, LRC, EFS, and FFDM in radically treated cervical cancer patients and should be used in clinical practice in assessing prognosis in these patients.

## 1. Introduction

According to the newest data, cervical cancer ranks fifth in terms of incidence and mortality worldwide (604,127 new cases and 341,831 deaths in 2020) [1]. An early diagnosis leads to better overall survival (OS) in cervical cancer patients. There are two available tests used for screening: the Papanicolaou test and the HPV test [2]. However, imaging modalities like transvaginal or transrectal ultrasound, magnetic resonance imaging (MRI), computed tomography (CT), and lately, positron emission tomography combined with computed tomography (PET/CT) are essential for adequate assessment of tumor size, invasiveness, and detection of distant metastases [3]. MRI and CT are widely used to detect metastatic lymph nodes based on their size and morphological features. PET/CT with the most commonly used radiotracer: glucose analogue labelled with fluorine-18 (2-deoxy-2-[^18^F]fluoro-D-glucose, [^18^F]FDG) has an advantage over these modalities and provides both: morphological and anatomical information [4]. Several qualitative and quantitative PET-derived parameters have been found to be prognostic in the pretreatment of cervical carcinoma and in assessing the recurrence and restaging of other gynaecological malignancies [5,6,7,8,9]. Maximum standardised uptake value (SUV_max_), total lesion glycolysis (TLG), and metabolic tumor volume (MTV) are the most common PET-derived parameters with proven significance in assessing therapy response and outcome in cervical cancer patients [10,11].

The aim of this retrospective study was to assess the predictive value of [^18^F]FDG PET-derived parameters in radically treated cervical cancer patients.

## 2. Results

A total of 402 patients underwent radiochemotherapy (CRT) as primary treatment. Those patients, who were treated with a hysterectomy at the beginning, were then stratified to adjuvant treatment according to their risk factors: either chemotherapy or radiotherapy or radiochemotherapy. Hyperthermia was given as combined treatment with radiotherapy and radiochemotherapy.

Univariate Cox regression using the metric PET parameters revealed that only MTV and TLG are significant prognostic factors for event free survival (EFS), overall survival (OS), and freedom from distant metastases (FFDM). Moreover, MTV was also a prognostic factor for locoregional control (LRC).

SUV_max_ and SUV_mean_ were not prognostic for any of the investigated endpoints, and were, therefore, excluded in further analysis.

From the clinical parameters, hyperthermia and brachytherapy were prognostic for EFS, OS, and LRC. FIGO stage above II showed a significant effect for EFS, OS, and FFDM. Moreover, hysterectomy was prognostic for OS and histology was prognostic for FFDM. No significant effect was found in CRT in all four endpoints. Results for all investigated parameters are listed in Table 1.

**Figure 1:**
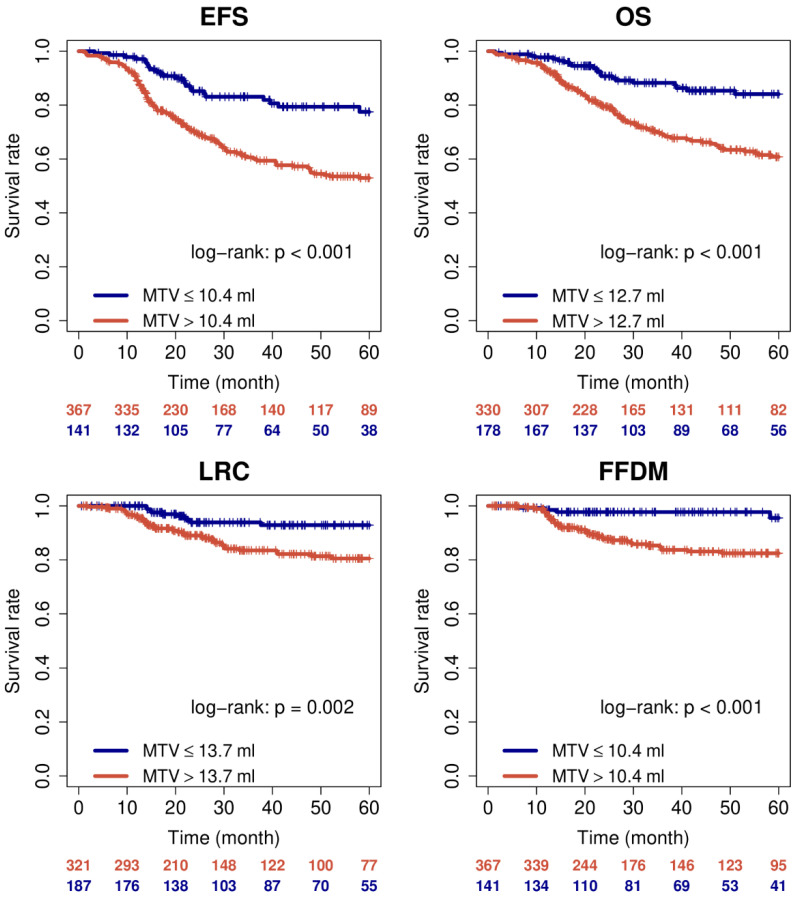
Kaplan-Meier curves for MTV with respect to EFS, OS, LRC, and FFDM.

As expected MTV and TLG were prognostic in univariate Cox regression also after binarisation. Corresponding Kaplan-Meier curves for MTV are shown in Figure 1.

Regression analysis revealed a notably higher hazard ratio for MTV than for TLG (Table 2).

Therefore, and due to the high correlation of MTV and TLG (*R*2 = 0.88, *p* < 0.001), only MTV was analysed in multivariate Cox regression together with the corresponding clinical parameters as confounding factors.

In this analysis MTV remained a prognostic factor for all four endpoints (Table 3) indicating its independent prognostic value.

The cutoff stability test performed for MTV revealed a wide range of cutoff values, leading to a significant effect for all four endpoints (Table 4, right). Cutoff values were also stable according to the bootstrap analysis (Table 4, left). However, this has to be confirmed in an independent patient group.

## 3. Discussion

Several quantitative and qualitative PET-derived parameters have been reported to be prognostic factors in cervical cancer [12,13]. The most common, SUV_max_, despite its confirmed prognostication value, might be affected by several factors (segmentation method, patient glucose level, reconstruction algorithm, etc.), and does not represent the whole tumor. To measure the metabolic activity in the whole tumor and its entirety, volume based parameters, such as MTV and TLG have become more of an object of interest lately [14]. Takagi et al. based on the analysis of 38 cervical cancer patients noted that SUV_max_ value of primary tumor is useful in differentiating between stage ≤I and ≥II [15]. Our analysis showed that neither SUV_max_ nor SUV_mean_ had any significance in all assessed endpoints. This might be caused by a notable difference in cohort groups between studies.

In their work on 91 patients, Sun et al. showed that cervical metabolic tumor volume (CMTV) above 53.75 mL significantly decreases OS in cervical cancer patients [5]. Additionally, in their univariate analysis they also observed that CMTV and cervical total lesion glycolysis (CTLG) were significant prognostic factors for OS in terms of FIGO stage, age, lymphadenopathy, and SUV_max_ value. In our analysis, we also observed that MTV is an independent PET-derived prognostic factor, for OS, as well as EFS, LRC, and FFDM. Different values for MTV obtained between our and the abovementioned data are probably caused by a significant difference in the number of analysed patients (91 vs. 508). Moreover, our study also revealed that several clinical factors such as FIGO stage, brachytherapy, hyperthermia, are predictors in cervical cancer patients, however only MTV was an independent predictor on all four endpoints.

In their work, Wong et al. found that [^18^F]FDG PET/CT is an accurate diagnostic tool in detecting the local or distant recurrence in cervical cancer patients with 82% sensitivity, 97% specificity and 92% accuracy for the local and 100%, 90% and 94% for the distant one [16]. Our study revealed that the FIGO stage, histology of the primary tumor and MTV are significantly associated with FFDM. Moreover, MTV and brachytherapy were also prognostic factors for LRC. Brachytherapy was documented in many studies to be the essential part of cervical cancer treatment leading to improved outcomes [17,18].

Wang et al. showed that TLG ≥113.4 mL and MTV ≥18.3 cm^3^ of primary cervical tumor were associated with worse DFS, DMFS, and OS [19]. No significance was noted in OS, DFS, LC or DMFS for SUV_max_ or SUV_mean_ values either in univariate or multivariate analysis. Our analysis on a larger cohort group showed similar results for commonly used metabolic parameters (SUV_max_ and SUV_mean_), as well as for the volumetric parameters. However, as the TLG is a product of a SUV_mean_ and MTV, and a strong correlation between these two parameters was found, only MTV was included in the analysis. MTV proved to be a significant PET-derived metabolic parameter which has an influence on OS, EFS, LRC, and FFDM in 508 analysed cervical cancer patients.

Han et al. performed a meta-analysis on 660 patients from 12 studies, during which they assessed the value of volume-based [^18^F]FDG PET/CT parameters in uterine cervical cancer [20]. They found that higher values for MTV and TLG are significantly associated with worse DFS, EFS and OS. Similar results were obtained in this study. Moreover, well known clinical parameters are shown to be prognostic in cervical cancer patients, but only MTV was an independent prognostic parameter. MTV value above 10 mL was associated with worse EFS and FFDM, while above 13 mL and 14 mL, with worse OS and LRC, respectively.

Even though this study was carried out as a retrospective and single-centre analysis, which might be a major limitation, to the best of our knowledge, it is performed on the largest group of patients with verified long-term outcomes. Moreover, the heterogeneity of the treatment methods might affect the obtained results, but these treatment options were used according to the stage of the disease at the time of initial diagnosis. Nevertheless, we are aware that results obtained in this study should be validated in a multicentre, prospective study.

## 4. Materials and Methods

### 4.1. Patient Characteristic

A retrospective analysis was performed on a group of 508 newly diagnosed cervical cancer patients. All patients were admitted to the Gynaecology and Radiotherapy Department with radical treatment intent between May 2009 and May 2020. A medical chart review was performed to obtain the recurrence, and data from the Greater Poland Cancer Registry were used to estimate the patients’ prognosis. The majority of patients were diagnosed at stage III (49.2%) or II (29.5%), and squamous cell carcinoma was the most common histological type (89.6%). Chemotherapy and radiotherapy (including teleradiotherapy and brachytherapy) were the most common therapy methods used in the analysed group. Detailed patient and tumor characteristics are shown in Table 5.

All patients provided their informed consent for the treatment and diagnostic imaging procedure and because of the retrospective character of this study, bioethical committee approval was waived.

### 4.2. [^18^F]FDG PET/CT Analysis

All patients underwent a hybrid [^18^F]FDG PET/CT scan prior to therapy. Scans were performed using Gemini TF TOF PET/CT scanner (Philips Healthcare, Best, The Netherlands). The scan ranged from the vertex to the mid-thigh according to the standard whole-body acquisition protocol used in our department. Patients were fasting for at least 6 h before the injection of 364 ± 75 MBq [^18^F]FDG and the acquisition started 60 ± 15 min (range: 45–75 min) after injection. First, a low-dose multislice CT scan was obtained using a 16-slice multidetector scanner (parameters: 100–250 mAs, 120 kV, slice thickness 5 mm). The PET scan was performed in 3D mode with an acquisition time of 1.30 min per bed position (8–12 bed positions) covering the same field as the CT scan. The obtained images were reconstructed using the ordered subset expectation maximisation (OSEM) iterative algorithm. SUV was normalized by body weight.

### 4.3. Data Analysis

The metabolically active part of the primary tumor was delineated in the PET data by an automatic algorithm based on adaptive thresholding. The algorithm iteratively determines the local background of a lesion and then applies a background-corrected threshold; more detailed information can be found in [21,22] for more details. The resulting region of interest (ROI) delineation was inspected visually by an experienced observer (who was blinded to patient outcomes) and manually corrected when this was deemed necessary. This was the case in 59 out of 508 patients as the algorithm delineated also parts of the bladder. For the delineated ROIs, the parameters: SUV_max_, SUV_mean_, metabolic tumor volume (MTV), and total lesion glycolysis (TLG calculated as MTV *×* SUV_mean_) were computed. ROI definition and analysis were performed using the ROVER software, version 3.0.XX (ABX, Radeberg, Germany).

### 4.4. Statistical Analysis

Survival analysis was performed with respect to event free survival (EFS), overall survival (OS), locoregional control (LRC), and freedom from distant metastases (FFDM) measured from the start of therapy to death and/or event. Patients who did not keep follow-up appointments and for whom information on survival or tumor status, therefore, was unavailable were censored at the date of the last follow-up. For EFS, any disease recurrence (loco-regional or distant) or death was classified as an event. The association of endpoints with clinical and quantitative PET parameters was analysed using univariate Cox proportional hazard regression in which the PET parameters were included as metric parameters. PET parameters showing a significant effect in this analysis were further analysed in a univariate Cox regression using binarised PET parameters. The cutoff values were calculated by minimising the *p*-value in univariate Cox regression, as described by Bütof et al. [23]. The optimal cutoff was determined separately for EFS, OS, LRC, and FFDM. The stability of optimal cutoff values was tested using the bootstrap method (random resampling with replacement, 10^5^ samples). For each sample, a univariate Cox regression was performed in which the same cutoff as in the original data was used to define high- and low-risk groups. Mean (sample averaged) HR and *p*-value were computed. The fraction of samples yielding *p* < 0.05 was determined. Furthermore, the range of cutoff values for which *p* remains below 0.05 in univariate analysis was determined by successively decreasing/increasing the cutoff (starting at the optimal cutoff) and repeating univariate Cox regression in the original patient group. The probability of survival was computed and rendered as Kaplan–Meier curves. The independence of parameters was analysed by a multivariate Cox regression.

Statistical significance was assumed at a *p*-value of less than 0.05. Statistical analysis was performed with the R language and environment for statistical computing version 4.1.1 [24].

## 5. Conclusions

Pretreatment MTV from the primary tumor is an independent prognostic factor for assessing overall survival, event-free survival, locoregional control and freedom from distant metastases in radically treated cervical cancer patients. Further investigations are needed to confirm these promising results.

## Figures and Tables

**Table 1:** Univariate Cox regression. PET parameters were included as metric parameters. For patient age the median was used as cutoff value.

	**EFS**			**OS**		
Parameter	HR	95% CI	*p*-Value	HR	95% CI	*p*-Value
Age > 57 y	0.85	0.63–1.15	0.29	0.93	0.65–1.34	0.71
Histology SCC	0.96	0.58–1.59	0.88	1.24	0.65–2.37	0.52
Grading > 2	1.19	0.81–1.76	0.37	1.35	0.86–2.12	0.19
Hyperthermia	0.6	0.44–0.82	0.001	0.5	0.35–0.73	<0.001
Chemotherapy	1.12	0.69–1.82	0.66	1.36	0.73–2.53	0.33
Teleradiotherapy	0.82	0.55–1.23	0.34	1.01	0.61–1.67	0.96
Brachytherapy	0.46	0.33–0.64	<0.001	0.45	0.31–0.67	<0.001
Hysterectomy CRT	0.75 0.79	0.43–1.29 0.55–1.13	0.3 0.19	0.41 0.86	0.18–0.94 0.56–1.33	0.035 0.5
FIGO stage > II	2.11	1.51–2.96	<0.001	2.33	1.54–3.5	<0.001
MTV	1.009	1.005–1.012	<0.001	1.009	1.005–1.013	<0.001
TLG	1	1–1.001	0.0077	1	1–1.001	0.039
SUV_max_	1.004	0.979–1.03	0.75	1	0.97–1.031	1
SUV_mean_	0.997	0.957–1.038	0.87	0.99	0.94–1.04	0.66
	LRC			FFDM		
Parameter	HR	95% CI	*p*-value	HR	95% CI	*p*-value
Age > 57 y	0.67	0.39–1.16	0.15	0.68	0.39–1.19	0.18
Histology SCC	1.43	0.52–3.95	0.49	0.4	0.2–0.78	0.0068
Grading > 2	0.78	0.35–1.72	0.54	1.8	0.96–3.37	0.069
Hyperthermia	0.56	0.33–0.96	0.037	1.02	0.59–1.77	0.95
Chemotherapy	1.05	0.45–2.46	0.9	1.28	0.51–3.22	0.6
Teleradiotherapy	0.59	0.31–1.13	0.11	1.3	0.56–3.06	0.54
Brachytherapy	0.32	0.18–0.55	<0.001	0.76	0.39–1.48	0.42
Hysterectomy CRT	1.68 0.72	0.82–3.43 0.39–1.34	0.16 0.3	0.32 1.1	0.08–1.31 0.54–2.26	0.11 0.79
FIGO stage > II	1.46	0.84–2.56	0.18	1.96	1.07–3.58	0.029
MTV	1.008	1.001–1.015	0.016	1.01	1–1.02	<0.001
TLG	1.001	1–1.001	0.056	1.001	1–1.001	0.042
SUV_max_	1.03	0.99–1.07	0.2	1.02	0.98–1.06	0.39
SUV_mean_	1.02	0.96–1.09	0.5	1.02	0.95–1.09	0.55

**Table 2:** Univariate Cox regression. PET parameters were included as binary parameters.

Parameter	Risk	HR	95% CI	*p* Value
EFS				
MTV	>10.4 mL	2.57	1.67–3.97	<0.001
TLG	>133 mL	1.85	1.35–2.54	<0.001
OS				
MTV	>12.7 mL	2.8	1.75–4.48	<0.001
TLG	>91.9 mL	2.16	1.42–3.3	<0.001
LRC				
MTV	>13.7 mL	2.82	1.42–5.61	0.003
FFDM				
MTV	>10.4 mL	5.04	1.82–13.99	0.002
TLG	>201 mL	2.26	1.3–3.94	0.004

**Table 5:** Patient and tumor characteristics.

Characteristics	Value
Age (years)	
Mean *±* SD	55 *±* 12
Median	57
Histology	
SCC	455 (89.6)
Adeno Ca	53 (10.4)
Grading	
n/a	140 (27.6)
G1	28 (5.5)
G2	251 (49.4)
G3	89 (17.5)
FIGO stage	
I	59 (11.6)
II	150 (29.5)
III	250 (49.2)
IV	49 (9.7)
Therapy	
Hyperthermia	264 (52)
Chemotherapy	445 (87.6)
Teleradiotherapy	425 (83.7)
Brachytherapy	396 (78)
Hysterectomy CRT	57 (11.2) 402 (79.1)

**Table 3:** Multivariate Cox regression. PET parameters were included as metric parameters.

	**EFS**			**OS**		
Parameter	HR	95% CI	*p*-Value	HR	95% CI	*p*-Value
Histology		–			–	
Hyperthermia	0.947	0.894–1	0.063	0.84	0.764–0.924	<0.001
Brachytherapy	0.382	0.268–0.543	<0.001	0.432	0.286–0.651	<0.001
FIGO stage	1.77	1.45–2.16	<0.001	1.95	1.54–2.46	<0.001
MTV	1.01	1–1.01	0.005	1.01	1–1.01	0.013
	LRC			FFDM		
Parameter	HR	95% CI	*p*-value	HR	95% CI	*p*-value
Histology		-			-	
Hyperthermia	1.02	0.932–1.11	0.68	3.07	1.56–6.03	0.001
Brachytherapy	0.303	0.169–0.546	<0.001	1.73	–	
FIGO stage		-		1.73	1.19–2.53	0.004
MTV	1.01	1–1.01	0.021	1.01	1–1.02	0.009

**Table 4:** Evaluation of bootstrap samples and cutoff range. Column 4 shows the fraction of bootstrap samples for which the same cutoff value leads to *p* < 0.05, respectively.

	Bootstrap			Cutoff Range *p* < 0.05		
Endpoint	Mean HR	Mean *p* Value	*p <* 0.05	Min. Cutoff	Opt. Cutoff	Max. Cutoff
EFS	2.7	<0.001	100%	3 mL	10.4 mL	102.5 mL
OS	2.9	<0.001	100%	3.2 mL	12.7 mL	23.9 mL
LRC	3.2	0.021	90%	10.1 mL	13.7 mL	23.2 mL
FFDM	6.6	0.007	98%	5.8 mL	10.4 mL	13.2 mL

## Data Availability

The data presented in this study are available in article.

## References

[1] Sung H., Ferlay J., Siegel R.L., Laversanne M., Soerjomataram I., Jemal A., Bray F. (2021). Global Cancer Statistics 2020: GLOBOCAN Estimates of Incidence and Mortality Worldwide for 36 Cancers in 185 Countries. CA Cancer J. Clin..

[2] Tsikouras P., Zervoudis S., Manav B., Tomara E., Iatrakis G., Romanidis C., Bothou A., Galazios G. (2016). Cervical cancer: Screening, diagnosis and staging. J. BUON.

[3] Haldorsen I.S., Lura N., Blaakær J., Fischerova D., Werner H.M.J. (2019). What Is the Role of Imaging at Primary Diagnostic Work-Up in Uterine Cervical Cancer?. Curr. Oncol. Rep..

[4] Liu B., Gao S., Li S. (2017). A Comprehensive Comparison of CT, MRI, Positron Emission Tomography or Positron Emission Tomography/CT, and Diffusion Weighted Imaging-MRI for Detecting the Lymph Nodes Metastases in Patients with Cervical Cancer: A Meta-Analysis Based on 67 Studies. Gynecol. Obstet. Invest..

[5] Sun Y., Lu P., Yu L. (2016). The Volume-metabolic Combined Parameters from (18)F-FDG PET/CT May Help Predict the Outcomes of Cervical Carcinoma. Acad. Radiol..

[6] Herrera F.G., Breuneval T., Prior J.O., Bourhis J., Ozsahin M. (2016). [(18)F]FDG-PET/CT metabolic parameters as useful prognostic factors in cervical cancer patients treated with chemo-radiotherapy. Radiat. Oncol..

[7] Narayanan P., Sahdev A. (2017). The role of ^18^F-FDG PET CT in common gynaecological malignancies. Br. J. Radiol..

[8] Albano D., Bonacina M., Savelli G., Ferro P., Busnardo E., Gianolli L., Camoni L., Giubbini R., Bertagna F. (2021). Clinical and prognostic ^18^F-FDG PET/CT role in recurrent vulvar cancer: A multicentric experience. Jpn. J. Radiol.

[9] Albano D., Zizioli V., Treglia G., Odicino F., Giubbini R., Bertagna F. (2019). Role of ^18^F-FDG PET/CT in restaging and follow-up of patients with uterine sarcomas. Rev. Esp. Med. Nucl. Imagen Mol. Engl. Ed..

[10] Yilmaz M., Adli M., Celen Z., Zincirkeser S., Dirier A. (2010). FDG PET-CT in cervical cancer: Relationship between primary tumor FDG uptake and metastatic potential. Nucl. Med. Commun..

[11] Kidd E.A., Siegel B.A., Dehdashti F., Grigsby P.W. (2007). The standardized uptake value for F-18 fluorodeoxyglucose is a sensitive predictive biomarker for cervical cancer treatment response and survival. Cancer.

[12] Pan L., Cheng J., Zhou M., Yao Z., Zhang J. (2012). The SUV_max_ (maximum standardized uptake value for F-18 fluorodeoxyglucose) and serum squamous cell carcinoma antigen (SCC-ag) function as prognostic biomarkers in patients with primary cervical cancer. J. Cancer Res. Clin. Oncol..

[13] Chung H.H., Kim J.W., Han K.H., Eo J.S., Kang K.W., Park N.H., Song Y.S., Chung J.K., Kang S.B. (2011). Prognostic value of metabolic tumor volume measured by FDG-PET/CT in patients with cervical cancer. Gynecol. Oncol..

[14] Moon S.H., Hyun S.H., Choi J.Y. (2013). Prognostic significance of volume-based PET parameters in cancer patients. Korean J. Radiol..

[15] Takagi H., Sakamoto J., Osaka Y., Shibata T., Fujita S., Sasagawa T. (2018). Usefulness of the maximum standardized uptake value for diagnosis and staging patients with cervical cancer undergoing positron emission tomography/computed tomography. Medicine.

[16] Wong T.Z., Jones E.L., Coleman R.E. (2004). Positron emission tomography with 2-deoxy-2-[(18)F]fluoro-D-glucose for evaluating local and distant disease in patients with cervical cancer. Mol. Imaging Biol..

[17] Sturdza A., Pötter R., Fokdal L.U., Haie-Meder C., Tan L.T., Mazeron R., Petric P., Šegedin B., Jurgenliemk-Schulz I.M., Nomden C. (2016). Image guided brachytherapy in locally advanced cervical cancer: Improved pelvic control and survival in RetroEMBRACE, a multicenter cohort study. Radiother. Oncol..

[18] Tanderup K., Lindegaard J.C., Kirisits C., Haie-Meder C., Kirchheiner K., de Leeuw A., Jürgenliemk-Schulz I., Van Limbergen E., Pötter R. (2016). Image Guided Adaptive Brachytherapy in cervix cancer: A new paradigm changing clinical practice and outcome. Radiother. Oncol..

[19] Wang D., Liu X., Wang W., Huo L., Pan Q., Ren X., Zhang F., Hu K. (2021). The role of the metabolic parameters of 18F-FDG PET/CT in patients with locally advanced cervical cancer. Front. Oncol..

[20] Han S., Kim H., Kim Y.J., Suh C.H., Woo S. (2018). Prognostic value of volume-based metabolic parameters of 18F-FDG PET/CT In uterine cervical cancer: A systematic review and meta-analysis. Am. J. Roentgenol..

[21] Hofheinz F., Pötzsch C., Oehme L., Beuthien-Baumann B., Steinbach J., Kotzerke J., van den Hoff J. (2012). Automatic volume de- lineation in oncological PET. Evaluation of a dedicated software tool and comparison with manual delineation in clinical data sets. Nuklearmedizin.

[22] Hofheinz F., Langner J., Petr J., Beuthien-Baumann B., Steinbach J., Kotzerke J., van den Hoff J. (2013). An automatic method for accurate volume delineation of heterogeneous tumors in PET. Med. Phys..

[23] Bütof R., Hofheinz F., Zöphel K., Stadelmann T., Schmollack J., Jentsch C., Kotzerke J., Baumann M., van den Hoff J. (2015). Prognostic Value of Prethera- peutic Tumor-to-Blood Standardized Uptake Ratio in Patients with Esophageal Carcinoma. J. Nucl. Med..

[24] R Core Team (2021). R: A Language and Environment for Statistical Computing. R Foundation for Statistical Computing.

